# 1-{3-(4-Chloro­phen­yl)-5-[4-(propan-2-yl)phen­yl]-4,5-di­hydro-1*H*-pyrazol-1-yl}propan-1-one

**DOI:** 10.1107/S1600536814013075

**Published:** 2014-06-11

**Authors:** B. Narayana, Vinutha V. Salian, Balladka K. Sarojini, Jerry P. Jasinski

**Affiliations:** a Department of Studies in Chemistry, Mangalore University, Mangalagangotri 574 199, India; b Department of Studies in Chemistry, Industrial Chemistry Section, Mangalore University, Mangalagangotri 574 199, India; c Department of Chemistry, Keene State College, 229 Main Street, Keene, NH 03435-2001, USA

## Abstract

In the title compound, C_21_H_23_ClN_2_O, the dihedral angle between the benzene rings is 83.2 (6)°, while the mean plane of the pyrazole ring [r.m.s. deviation = 0.043 (1) Å] makes dihedral angles of 3.4 (3) and 86.2 (1)° with the benzene rings. In the crystal, a pair of weak C—H⋯O inter­actions between the benzene ring and the propan-1-one group link the mol­ecules into an inversion dimer with an *R*_2_^2^(16) graph-set motif. In addition, a weak π–π stacking inter­action [centroid–centroid distance = 3.959 (4) Å] connects the dimers into a tape running along [201].

## Related literature   

For the biological activity of pyrazolines, see: Taylor *et al.* (1992 ▶); Lombardino & Otterness (1977 ▶); Manna *et al.* (2005 ▶); Samshuddin *et al.* (2012*a* ▶,*b* ▶). For standard bond lengths, see: Allen *et al.* (1987 ▶). For a related structure, see: Narayana *et al.*, (2014 ▶).
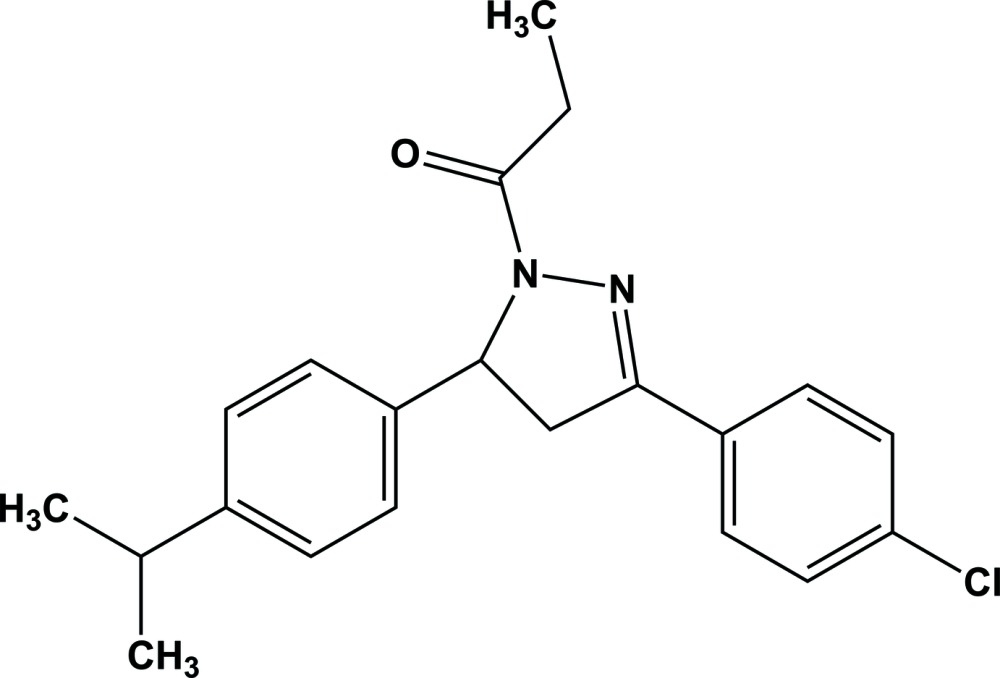


## Experimental   

### 

#### Crystal data  

C_21_H_23_ClN_2_O
*M* *_r_* = 354.86
Triclinic, 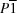

*a* = 6.6042 (3) Å
*b* = 10.1188 (9) Å
*c* = 14.4806 (12) Å
α = 98.444 (7)°
β = 90.650 (6)°
γ = 106.542 (6)°
*V* = 916.13 (12) Å^3^
*Z* = 2
Cu *K*α radiation
μ = 1.92 mm^−1^
*T* = 173 K
0.36 × 0.28 × 0.16 mm

#### Data collection  

Agilent Eos Gemini diffractometer
Absorption correction: multi-scan (*CrysAlis PRO* and *CrysAlis RED*; Agilent, 2012 ▶) *T*_min_ = 0.583, *T*_max_ = 0.736
5565 measured reflections
3519 independent reflections
3170 reflections with *I* > 2σ(*I*)
*R*_int_ = 0.039

#### Refinement  

*R*[*F* ^2^ > 2σ(*F* ^2^)] = 0.047
*wR*(*F* ^2^) = 0.133
*S* = 1.05
3519 reflections
230 parameters
H-atom parameters constrained
Δρ_max_ = 0.31 e Å^−3^
Δρ_min_ = −0.27 e Å^−3^

### 

Data collection: *CrysAlis PRO* (Agilent, 2012 ▶); cell refinement: *CrysAlis PRO* (Agilent, 2012 ▶); data reduction: *CrysAlis RED* (Agilent, 2012 ▶); program(s) used to solve structure: *SUPERFLIP* (Palatinus *et al.*, 2012 ▶); program(s) used to refine structure: *SHELXL2012* (Sheldrick, 2008 ▶); molecular graphics: *OLEX2* (Dolomanov *et al.*, 2009 ▶); software used to prepare material for publication: *OLEX2* (Dolomanov *et al.*, 2009 ▶).

## Supplementary Material

Crystal structure: contains datablock(s) I. DOI: 10.1107/S1600536814013075/is5364sup1.cif

Structure factors: contains datablock(s) I. DOI: 10.1107/S1600536814013075/is5364Isup2.hkl

Supporting information file. DOI: 10.1107/S1600536814013075/is5364Isup3.cml

CCDC reference: 1006823

Additional supporting information:  crystallographic information; 3D view; checkCIF report

## Figures and Tables

**Table 1:** Hydrogen-bond geometry (Å, °)

*D*—H⋯*A*	*D*—H	H⋯*A*	*D*⋯*A*	*D*—H⋯*A*
C11—H11⋯O1^i^	0.95	2.51	3.419 (2)	161

Symmetry code: (i) 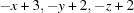.

**Fig. 1.**
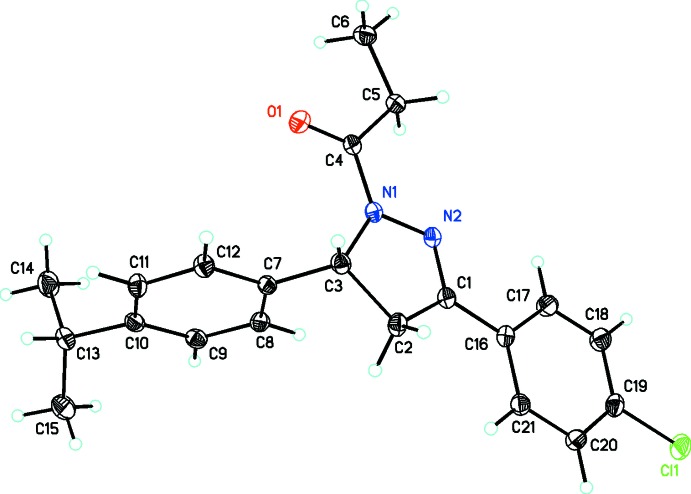
ORTEP drawing of the title compound showing the labeling scheme with 30% probability displacement ellipsoids.

**Fig. 2.**
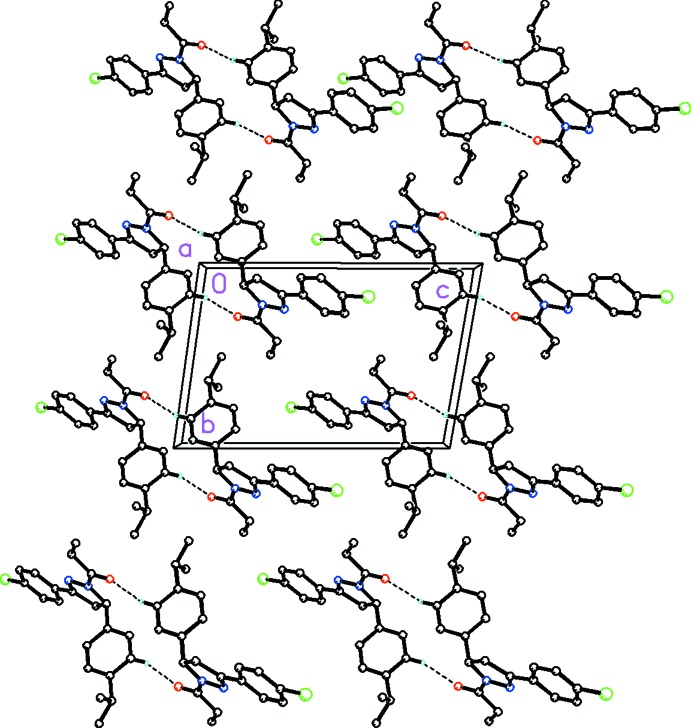
Molecular packing of the title compound viewed along the a axis. Dashed lines indicate weak C—H···O intermolecular interactions between the benzene ring and the ketone group forming dimers with an R22[16] ring motif. H atoms not involved in these interactions have been removed for clarity.

**Fig. 3.**
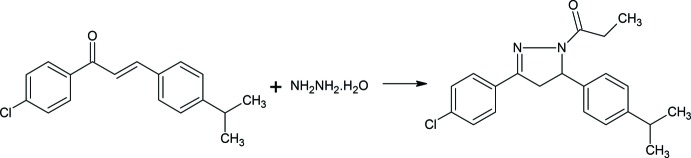
Synthesis of the title compound.

## supplementary crystallographic information

### S1. Comment

Pyrazolines are important nitrogen containing five-membered heterocyclic compounds. Pyrazoline derivatives possess important biological activities, including antitumor (Taylor *et al.*, 1992), immunosuppressive (Lombardino & Otterness, 1977), anticancer (Manna *et al.*, 2005), antimicrobial, analgesic and antioxidant activities (Samshuddin *et al.*, 2012*a*,*b*).

In the title compound, the dihedral angle between the benzene rings is 83.2 (6)°, while the pyrazole ring is separated from each of the benzene rings by 3.4 (3)° (C16–C21) and 86.2 (1)° (C7–C12), respectively (Fig. 1). Bond lengths are in normal ranges (Allen *et al.*, 1987). In the crystal, a weak C—H···O intermolecular interaction between the benzene ring and the propan-1-one group is observed forming dimers in an *R*_2_^2^(16) ring-set motif (Fig. 2). In addition, a weak π–π intermolecular stacking interaction [*Cg*3···*Cg*3 (1 - *x*, 2 - *y*, 1 - *z*) = 3.959 (4) Å; *Cg*3: C16–C21] is present and influences the crystal packing.

### S2. Experimental

To a mixture of (2*E*)-1-(4-chlorophenyl)-3-[4-(propan-2-yl)phenyl] prop-2-en-1-one (2.85 g, 0.01 mol) and hydrazine hydrate (0.5 mL, 0.01 mol) in 20 mL propionic acid was refluxed for 8 h (Fig. 3). The reaction mixture was cooled and poured into 50 mL ice-cold water. The precipitate formed was collected by filtration and purified by recrystallization from ethanol. Single crystals were grown from DMF by the slow evaporation method. (m.p.: 365–367 K).

### S3. Refinement

All of the H atoms were placed in their calculated positions and then refined using the riding model with Atom—H lengths of 0.95–1.00 Å (CH), 0.99 Å (CH_2_) or 0.98 Å (CH_3_). Isotropic displacement parameters for these atoms were set to 1.2 (CH, CH_2_) or 1.5 (CH_3_) times *U*_eq_ of the parent atom. Idealised Me refined as a rotating group.

### Crystal data

**Table:**

C_21_H_23_ClN_2_O	*Z* = 2
*M**_r_* = 354.86	*F*(000) = 376
Triclinic, *P*1	*D*_x_ = 1.286 Mg m^−^^3^
*a* = 6.6042 (3) Å	Cu *K*α radiation, λ = 1.54184 Å
*b* = 10.1188 (9) Å	Cell parameters from 2712 reflections
*c* = 14.4806 (12) Å	θ = 4.6–72.1°
α = 98.444 (7)°	µ = 1.92 mm^−^^1^
β = 90.650 (6)°	*T* = 173 K
γ = 106.542 (6)°	Irregular, colourless
*V* = 916.13 (12) Å^3^	0.36 × 0.28 × 0.16 mm

### Data collection

**Table:**

Agilent Eos Gemini diffractometer	3519 independent reflections
Radiation source: Enhance (Cu) X-ray Source	3170 reflections with *I* > 2σ(*I*)
Graphite monochromator	*R*_int_ = 0.039
Detector resolution: 16.0416 pixels mm^-1^	θ_max_ = 72.2°, θ_min_ = 4.6°
ω scans	*h* = −6→8
Absorption correction: multi-scan (*CrysAlis PRO* and *CrysAlis RED*; Agilent, 2012)	*k* = −12→12
*T*_min_ = 0.583, *T*_max_ = 0.736	*l* = −14→17
5565 measured reflections	

### Refinement

**Table:**

Refinement on *F*^2^	Hydrogen site location: inferred from neighbouring sites
Least-squares matrix: full	H-atom parameters constrained
*R*[*F*^2^ > 2σ(*F*^2^)] = 0.047	*w* = 1/[σ^2^(*F*_o_^2^) + (0.0778*P*)^2^ + 0.2126*P*] where *P* = (*F*_o_^2^ + 2*F*_c_^2^)/3
*wR*(*F*^2^) = 0.133	(Δ/σ)_max_ < 0.001
*S* = 1.05	Δρ_max_ = 0.31 e Å^−^^3^
3519 reflections	Δρ_min_ = −0.27 e Å^−^^3^
230 parameters	Extinction correction: *SHELXL2012* (Sheldrick, 2008), Fc^*^=kFc[1+0.001xFc^2^λ^3^/sin(2θ)]^-1/4^
0 restraints	Extinction coefficient: 0.0079 (10)
Primary atom site location: structure-invariant direct methods	

### Special details

**Table:**

Geometry. All esds (except the esd in the dihedral angle between two l.s. planes) are estimated using the full covariance matrix. The cell esds are taken into account individually in the estimation of esds in distances, angles and torsion angles; correlations between esds in cell parameters are only used when they are defined by crystal symmetry. An approximate (isotropic) treatment of cell esds is used for estimating esds involving l.s. planes.

### Fractional atomic coordinates and isotropic or equivalent isotropic displacement parameters (Å^2^)

**Table:**

	*x*	*y*	*z*	*U*_iso_*/*U*_eq_	
Cl1	0.09015 (7)	0.80472 (5)	0.39149 (3)	0.04238 (19)	
O1	1.2342 (2)	0.71922 (13)	0.84127 (9)	0.0348 (3)	
N1	0.9973 (2)	0.77738 (13)	0.75592 (9)	0.0247 (3)	
N2	0.8703 (2)	0.75374 (13)	0.67523 (9)	0.0239 (3)	
C1	0.7420 (2)	0.82811 (16)	0.68834 (11)	0.0234 (3)	
C2	0.7635 (2)	0.91195 (18)	0.78502 (11)	0.0272 (4)	
H2A	0.6352	0.8793	0.8198	0.033*	
H2B	0.7900	1.0126	0.7822	0.033*	
C3	0.9569 (2)	0.88334 (16)	0.83017 (11)	0.0243 (3)	
H3	0.9150	0.8405	0.8876	0.029*	
C4	1.1291 (2)	0.69830 (16)	0.76809 (11)	0.0249 (3)	
C5	1.1314 (3)	0.58555 (16)	0.68676 (12)	0.0280 (4)	
H5A	0.9900	0.5161	0.6769	0.034*	
H5B	1.1619	0.6280	0.6292	0.034*	
C6	1.2960 (3)	0.51247 (19)	0.70421 (14)	0.0363 (4)	
H6A	1.2959	0.4423	0.6498	0.054*	
H6B	1.2625	0.4668	0.7596	0.054*	
H6C	1.4361	0.5811	0.7145	0.054*	
C7	1.1482 (2)	1.00998 (15)	0.85394 (10)	0.0219 (3)	
C8	1.2222 (3)	1.10049 (17)	0.78993 (11)	0.0258 (3)	
H8	1.1504	1.0837	0.7302	0.031*	
C9	1.3995 (3)	1.21478 (17)	0.81238 (11)	0.0271 (3)	
H9	1.4486	1.2740	0.7672	0.033*	
C10	1.5071 (3)	1.24473 (16)	0.89944 (11)	0.0252 (3)	
C11	1.4312 (3)	1.15487 (17)	0.96358 (11)	0.0278 (4)	
H11	1.5005	1.1732	1.0239	0.033*	
C12	1.2564 (3)	1.03921 (17)	0.94090 (11)	0.0262 (3)	
H12	1.2095	0.9787	0.9856	0.031*	
C13	1.7049 (3)	1.36693 (17)	0.92541 (12)	0.0297 (4)	
H13	1.7213	1.3878	0.9952	0.036*	
C14	1.9003 (3)	1.3267 (2)	0.89069 (16)	0.0432 (5)	
H14A	1.8869	1.3024	0.8224	0.065*	
H14B	1.9124	1.2463	0.9183	0.065*	
H14C	2.0270	1.4056	0.9091	0.065*	
C15	1.6934 (3)	1.49961 (19)	0.89028 (16)	0.0450 (5)	
H15A	1.8187	1.5766	0.9146	0.067*	
H15B	1.5659	1.5228	0.9119	0.067*	
H15C	1.6884	1.4847	0.8218	0.067*	
C16	0.5854 (2)	0.82406 (16)	0.61440 (11)	0.0245 (3)	
C17	0.5637 (3)	0.73309 (19)	0.52963 (12)	0.0315 (4)	
H17	0.6547	0.6753	0.5189	0.038*	
C18	0.4115 (3)	0.7264 (2)	0.46142 (12)	0.0338 (4)	
H18	0.3968	0.6641	0.4042	0.041*	
C19	0.2804 (3)	0.81191 (18)	0.47760 (12)	0.0301 (4)	
C20	0.2989 (3)	0.90336 (18)	0.56024 (12)	0.0294 (4)	
H20	0.2088	0.9619	0.5702	0.035*	
C21	0.4514 (2)	0.90838 (17)	0.62857 (11)	0.0272 (4)	
H21	0.4644	0.9703	0.6858	0.033*	

### Atomic displacement parameters (Å^2^)

**Table:**

	*U*^11^	*U*^22^	*U*^33^	*U*^12^	*U*^13^	*U*^23^
Cl1	0.0387 (3)	0.0443 (3)	0.0417 (3)	0.0081 (2)	−0.0167 (2)	0.0083 (2)
O1	0.0367 (7)	0.0342 (7)	0.0332 (7)	0.0118 (5)	−0.0104 (5)	0.0020 (5)
N1	0.0265 (7)	0.0227 (6)	0.0223 (6)	0.0062 (5)	−0.0045 (5)	−0.0024 (5)
N2	0.0236 (7)	0.0228 (6)	0.0230 (6)	0.0046 (5)	−0.0026 (5)	0.0009 (5)
C1	0.0214 (7)	0.0229 (7)	0.0230 (7)	0.0026 (6)	0.0013 (6)	0.0021 (6)
C2	0.0218 (8)	0.0321 (8)	0.0249 (8)	0.0072 (6)	−0.0007 (6)	−0.0033 (6)
C3	0.0251 (8)	0.0242 (7)	0.0219 (7)	0.0063 (6)	0.0014 (6)	−0.0002 (6)
C4	0.0228 (7)	0.0204 (7)	0.0287 (8)	0.0024 (6)	−0.0017 (6)	0.0032 (6)
C5	0.0273 (8)	0.0223 (7)	0.0333 (9)	0.0075 (6)	−0.0015 (6)	0.0004 (6)
C6	0.0329 (9)	0.0277 (8)	0.0497 (11)	0.0117 (7)	0.0013 (8)	0.0043 (7)
C7	0.0226 (7)	0.0218 (7)	0.0209 (7)	0.0079 (6)	0.0004 (6)	−0.0012 (5)
C8	0.0306 (8)	0.0272 (8)	0.0198 (7)	0.0101 (6)	−0.0031 (6)	0.0019 (6)
C9	0.0336 (9)	0.0234 (7)	0.0253 (8)	0.0085 (6)	0.0030 (6)	0.0066 (6)
C10	0.0262 (8)	0.0210 (7)	0.0278 (8)	0.0074 (6)	0.0019 (6)	0.0006 (6)
C11	0.0286 (8)	0.0287 (8)	0.0224 (8)	0.0043 (7)	−0.0036 (6)	0.0010 (6)
C12	0.0291 (8)	0.0271 (8)	0.0206 (7)	0.0051 (6)	0.0009 (6)	0.0040 (6)
C13	0.0317 (9)	0.0237 (8)	0.0287 (8)	0.0022 (7)	0.0000 (7)	0.0005 (6)
C14	0.0292 (9)	0.0395 (10)	0.0530 (12)	0.0027 (8)	−0.0010 (8)	−0.0028 (9)
C15	0.0456 (11)	0.0261 (9)	0.0575 (13)	0.0008 (8)	−0.0035 (9)	0.0079 (8)
C16	0.0213 (7)	0.0266 (8)	0.0234 (8)	0.0037 (6)	−0.0003 (6)	0.0036 (6)
C17	0.0333 (9)	0.0350 (9)	0.0269 (8)	0.0139 (7)	−0.0009 (7)	−0.0013 (7)
C18	0.0358 (9)	0.0380 (9)	0.0260 (8)	0.0115 (7)	−0.0040 (7)	−0.0014 (7)
C19	0.0262 (8)	0.0321 (9)	0.0296 (8)	0.0028 (7)	−0.0047 (6)	0.0085 (7)
C20	0.0251 (8)	0.0291 (8)	0.0351 (9)	0.0089 (6)	0.0004 (7)	0.0067 (7)
C21	0.0244 (8)	0.0268 (8)	0.0284 (8)	0.0056 (6)	0.0014 (6)	0.0012 (6)

### Geometric parameters (Å, º)

**Table:**

Cl1—C19	1.7391 (17)	C9—C10	1.389 (2)
O1—C4	1.218 (2)	C10—C11	1.393 (2)
N1—N2	1.3812 (18)	C10—C13	1.520 (2)
N1—C3	1.4858 (19)	C11—H11	0.9500
N1—C4	1.365 (2)	C11—C12	1.386 (2)
N2—C1	1.282 (2)	C12—H12	0.9500
C1—C2	1.509 (2)	C13—H13	1.0000
C1—C16	1.468 (2)	C13—C14	1.529 (3)
C2—H2A	0.9900	C13—C15	1.525 (2)
C2—H2B	0.9900	C14—H14A	0.9800
C2—C3	1.546 (2)	C14—H14B	0.9800
C3—H3	1.0000	C14—H14C	0.9800
C3—C7	1.515 (2)	C15—H15A	0.9800
C4—C5	1.518 (2)	C15—H15B	0.9800
C5—H5A	0.9900	C15—H15C	0.9800
C5—H5B	0.9900	C16—C17	1.401 (2)
C5—C6	1.516 (2)	C16—C21	1.391 (2)
C6—H6A	0.9800	C17—H17	0.9500
C6—H6B	0.9800	C17—C18	1.382 (2)
C6—H6C	0.9800	C18—H18	0.9500
C7—C8	1.393 (2)	C18—C19	1.388 (3)
C7—C12	1.390 (2)	C19—C20	1.382 (3)
C8—H8	0.9500	C20—H20	0.9500
C8—C9	1.387 (2)	C20—C21	1.389 (2)
C9—H9	0.9500	C21—H21	0.9500
			
N2—N1—C3	113.63 (12)	C9—C10—C13	122.90 (14)
C4—N1—N2	121.84 (13)	C11—C10—C13	119.59 (15)
C4—N1—C3	124.15 (13)	C10—C11—H11	119.4
C1—N2—N1	108.33 (12)	C12—C11—C10	121.14 (15)
N2—C1—C2	114.20 (14)	C12—C11—H11	119.4
N2—C1—C16	120.84 (14)	C7—C12—H12	119.4
C16—C1—C2	124.92 (14)	C11—C12—C7	121.25 (14)
C1—C2—H2A	111.2	C11—C12—H12	119.4
C1—C2—H2B	111.2	C10—C13—H13	107.4
C1—C2—C3	102.65 (13)	C10—C13—C14	110.30 (14)
H2A—C2—H2B	109.2	C10—C13—C15	113.48 (15)
C3—C2—H2A	111.2	C14—C13—H13	107.4
C3—C2—H2B	111.2	C15—C13—H13	107.4
N1—C3—C2	100.81 (12)	C15—C13—C14	110.46 (16)
N1—C3—H3	109.6	C13—C14—H14A	109.5
N1—C3—C7	112.12 (12)	C13—C14—H14B	109.5
C2—C3—H3	109.6	C13—C14—H14C	109.5
C7—C3—C2	114.82 (13)	H14A—C14—H14B	109.5
C7—C3—H3	109.6	H14A—C14—H14C	109.5
O1—C4—N1	120.11 (15)	H14B—C14—H14C	109.5
O1—C4—C5	123.92 (14)	C13—C15—H15A	109.5
N1—C4—C5	115.96 (14)	C13—C15—H15B	109.5
C4—C5—H5A	109.3	C13—C15—H15C	109.5
C4—C5—H5B	109.3	H15A—C15—H15B	109.5
H5A—C5—H5B	108.0	H15A—C15—H15C	109.5
C6—C5—C4	111.61 (14)	H15B—C15—H15C	109.5
C6—C5—H5A	109.3	C17—C16—C1	120.97 (14)
C6—C5—H5B	109.3	C21—C16—C1	120.24 (14)
C5—C6—H6A	109.5	C21—C16—C17	118.76 (15)
C5—C6—H6B	109.5	C16—C17—H17	119.6
C5—C6—H6C	109.5	C18—C17—C16	120.78 (16)
H6A—C6—H6B	109.5	C18—C17—H17	119.6
H6A—C6—H6C	109.5	C17—C18—H18	120.5
H6B—C6—H6C	109.5	C17—C18—C19	119.09 (16)
C8—C7—C3	121.51 (14)	C19—C18—H18	120.5
C12—C7—C3	120.69 (14)	C18—C19—Cl1	119.18 (14)
C12—C7—C8	117.80 (14)	C20—C19—Cl1	119.38 (14)
C7—C8—H8	119.6	C20—C19—C18	121.45 (16)
C9—C8—C7	120.73 (14)	C19—C20—H20	120.5
C9—C8—H8	119.6	C19—C20—C21	118.94 (15)
C8—C9—H9	119.2	C21—C20—H20	120.5
C8—C9—C10	121.58 (14)	C16—C21—H21	119.5
C10—C9—H9	119.2	C20—C21—C16	120.97 (15)
C9—C10—C11	117.48 (15)	C20—C21—H21	119.5
			
Cl1—C19—C20—C21	−179.97 (12)	C3—C7—C8—C9	−179.03 (14)
O1—C4—C5—C6	−5.7 (2)	C3—C7—C12—C11	−179.83 (14)
N1—N2—C1—C2	−1.75 (18)	C4—N1—N2—C1	170.50 (14)
N1—N2—C1—C16	−179.56 (13)	C4—N1—C3—C2	−167.46 (14)
N1—C3—C7—C8	66.98 (18)	C4—N1—C3—C7	69.93 (19)
N1—C3—C7—C12	−112.81 (16)	C7—C8—C9—C10	−1.2 (2)
N1—C4—C5—C6	175.14 (14)	C8—C7—C12—C11	0.4 (2)
N2—N1—C3—C2	5.53 (16)	C8—C9—C10—C11	0.5 (2)
N2—N1—C3—C7	−117.09 (14)	C8—C9—C10—C13	178.43 (15)
N2—N1—C4—O1	−177.75 (14)	C9—C10—C11—C12	0.7 (2)
N2—N1—C4—C5	1.4 (2)	C9—C10—C13—C14	−83.6 (2)
N2—C1—C2—C3	5.09 (18)	C9—C10—C13—C15	40.9 (2)
N2—C1—C16—C17	3.7 (2)	C10—C11—C12—C7	−1.1 (3)
N2—C1—C16—C21	−178.10 (14)	C11—C10—C13—C14	94.32 (19)
C1—C2—C3—N1	−5.77 (15)	C11—C10—C13—C15	−141.14 (17)
C1—C2—C3—C7	114.94 (14)	C12—C7—C8—C9	0.8 (2)
C1—C16—C17—C18	177.91 (16)	C13—C10—C11—C12	−177.35 (15)
C1—C16—C21—C20	−178.43 (14)	C16—C1—C2—C3	−177.20 (14)
C2—C1—C16—C17	−173.90 (16)	C16—C17—C18—C19	0.4 (3)
C2—C1—C16—C21	4.3 (2)	C17—C16—C21—C20	−0.1 (2)
C2—C3—C7—C8	−47.29 (19)	C17—C18—C19—Cl1	179.47 (14)
C2—C3—C7—C12	132.92 (15)	C17—C18—C19—C20	0.0 (3)
C3—N1—N2—C1	−2.67 (17)	C18—C19—C20—C21	−0.5 (3)
C3—N1—C4—O1	−5.3 (2)	C19—C20—C21—C16	0.6 (2)
C3—N1—C4—C5	173.88 (13)	C21—C16—C17—C18	−0.4 (3)

### Hydrogen-bond geometry (Å, º)

**Table:**

*D*—H···*A*	*D*—H	H···*A*	*D*···*A*	*D*—H···*A*
C11—H11···O1^i^	0.95	2.51	3.419 (2)	161

Symmetry code: (i) −*x*+3, −*y*+2, −*z*+2.
